# Anti-Inflammatory and Antinociceptive Properties of the Quercetin-3-Oleate AV2, a Novel FFAR1 Partial Agonist

**DOI:** 10.3390/ijms252111635

**Published:** 2024-10-30

**Authors:** Federica Pessina, Ilenia Casini, Alessandra Gamberucci, Gabriele Carullo, Cinzia Signorini, Antonella Brizzi, Francesca Aiello, Anna Maria Aloisi, Stefano Pieretti

**Affiliations:** 1 Department of Molecular and Developmental Medicine, University of Siena, Via A. Moro 2, 53100 Siena, Italy; alessandra.gamberucci@unisi.it (A.G.); cinzia.signorini@unisi.it (C.S.); 2 Department of Medicine, Surgery and Neuroscience, University of Siena, Via A. Moro 2, 53100 Siena, Italy; ilenia.casini@student.unisi.it (I.C.); annamaria.aloisi@unisi.it (A.M.A.); 3 Department of Biotechnology, Chemistry and Pharmacy, University of Siena, Via A. Moro 2, 53100 Siena, Italy; gabriele.carullo@unisi.it; 4 Department of Pharmacy, Health and Nutritional Sciences, University of Calabria, 87036 Arcavacata, Italy; francesca.aiello@unical.it; 5 Istituto Superiore di Sanità, National Centre for Drug Research and Evaluation, Viale Regina Elena 299, 00161 Rome, Italy; stefano.pieretti@iss.it

**Keywords:** quercetin-3-oleate (AV2), inflammation, nociception, oxidative stress, FFAR1

## Abstract

Free fatty acid receptor 1 (FFAR1) has emerged as the most targeted isoform of the free fatty acid receptors because of its involvement in the modulation of energy balance and its potential role in the control of inflammatory and pain conditions. Quercetin-3-oleate (AV2), recognized as a new FFAR1 partial agonist, was investigated for its ability to modulate inflammation and nociception. Human immortal neuroblastoma SH and the murine macrophagic RAW 264.7 cells were used to evaluate cell viability, the potential cytoprotective activity, and the anti-inflammatory properties of AV2 in vitro. Paw edema, caused by zymosan-A, and the formalin test were used to assess the in vivo anti-inflammatory and antinociceptive effects in CD-1 mice. In vitro, AV2 was devoid of cytotoxicity, significantly reduced ROS in both cell types, and protected RAW 264.7 cells from lipopolysaccharide damage by reducing tumor necrosis factor-α production. Interestingly, AV2 induced a transient elevation of intracellular calcium that was reduced in cells, pre-incubated with the FFAR1 antagonist DC260126. In vivo, AV2 reduced formalin-induced nociception and zymosan A-induced paw edema, and both effects were reversed by the FFAR1 antagonist GW1100. In conclusion, these data strongly support the AV2-mediated antioxidant, anti-inflammatory, and antinociceptive activity. AV2 represents a promising molecule for the clinical management of inflammatory-related pain conditions.

## 1. Introduction

Free fatty acids (FFAs) are essential components of cell membranes. At the same time, they play a key role as mediators since they activate a signaling cascade involving peculiar G protein-coupled receptors (GPCRs), known as free fatty acid receptors (FFARs). Among the four FFARs identified, long-chain fatty acids interact with FFARs type 1 and type 4, whereas short-chain fatty acids interact with FFARs type 2 and type 3, and their activation participates in the regulation of multiple biological processes, such as metabolic and energetic homeostasis, as well as immune responses [1,2]. Therefore, FFARs have rapidly gained attention as attractive therapeutic targets for diabetes, obesity, and metabolic syndromes, prompting drug discovery studies [3,4].

**Figure 1:**
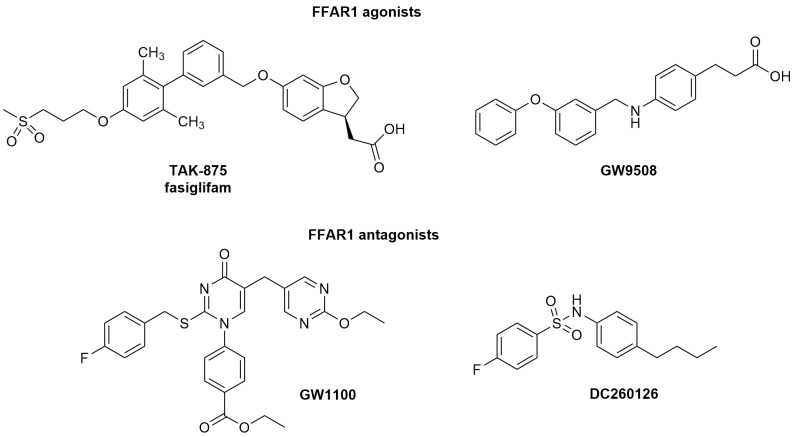
Chemical structure of FFAR1 agonists and antagonists.

FFAR1 has emerged as the most targeted isoform of this family, and its involvement in the regulation of energy balance has been intensely investigated [5,6]. Nevertheless, this receptor is expressed not only in pancreatic β-cells and enteroendocrine cells but also in the central nervous system (CNS) [7] and in different cell types involved in inflammation, such as microglia, fibroblasts, and macrophages [8], highlighting its potential role in the control of inflammatory and pain conditions [9]. In keratinocytes, the activation of FFAR1 receptors by GW9508 (Figure 1), an FFAR1 and FFAR4 agonist, reduced the release of several chemokines, such as CCL5 and CCL17 (C-C motif chemokine ligands 5 and 17), and attenuated skin immune inflammation [10]. Docosahexaenoic acid (DHA), a long-chain fatty acid endowed with FFAR1 agonist activity, showed analgesic effects, suppressing inflammatory-related pain such as that associated with joint pain [11], rheumatoid arthritis [12], and headaches [13]. Nakamoto et al. [14] reported that intracerebroventricular (i.c.v.) administration of DHA or GW9508 significantly suppressed formalin-induced pain. Moreover, both compounds also reduced complete Freund’s adjuvant-induced mechanical allodynia and thermal hyperalgesia through hypothalamic FFAR1 activation. Astrocytes seemed to participate in the regulation of the FFAR1-induced antinociception by increasing the release of FFAs and, consequently, FFAR1 expression [15].

Taken together, these findings show a prospective novel therapeutic approach for the development of new drugs that could control and relieve inflammatory and pain conditions.

Over the years, several FFAR1 ligands endowed with antagonist and partial/full agonist activity have been identified (Figure 1). Among these, only the partial agonist TAK-875/fasiglifam and JTT-851, a Japan tobacco derivative (unpublished structure), reached late phase III and phase II clinical trials, respectively [16]. However, side effects did not allow the trials to proceed, highlighting the need to identify more effective and safer FFAR1 candidates. In this context, our group has identified oleic acyl derivatives as FFAR1 ligands by combining two dietary compounds, such as quercetin and oleic acid [17].

In fact, selective acylation of quercetin phenolic hydroxyls with natural acids represented a valid approach to modulate its biological activity and overcome its poor bioavailability [18]. Accordingly, oleic acid is a valuable acyl-donor, as it is an edible and safe nutrient, endowed with antioxidant, cholesterol, and blood pressure-regulatory activity.

**Figure 2:**
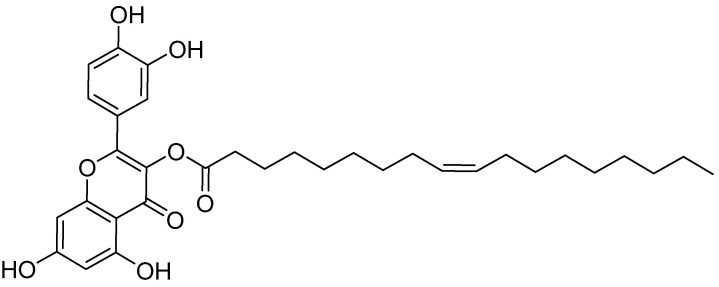
Chemical structure of the quercetin-3-oleoyl derivative AV2.

The quercetin-3-oleoyl derivative AV2 (Figure 2, see Tables S1 and S2 in Supplementary Material) was regio-selectively obtained simply by adding pancreatic porcine lipase (PPL) to a solution of quercetin and oleic acid in acetone at 37 °C. This green method allowed for the production of AV2, which retains the *Z* configuration of the initial oleic acid, in a very high yield, recycling the enzyme at least five times [19]. AV2 behaves as an FFAR1 partial agonist endowed with secretagogue activity. Our group already demonstrated its ability to induce insulin secretion from INS-1 832/13 β-cells and a wound-healing effect with synergistic effects compared to quercetin and oleic acid alone [20].

This study aimed to explore the physio-pharmacological properties of AV2 by evaluating its potential anti-inflammatory and antinociceptive effects due to the interaction with FFAR1. Therefore, cell viability, protective effects of AV2 against oxidative stress damage, and anti-inflammatory activity were evaluated in in vitro assays, whereas well-characterized experimental models of inflammation and pain in vivo were adopted, using as the positive control the FFAR1 agonist GW9508, and as the antagonist compound, GW1100 (Figure 1).

## 2. Results

### 2.1. In Vitro Experiments

#### 2.1.1. Cell Viability

**Figure 3:**
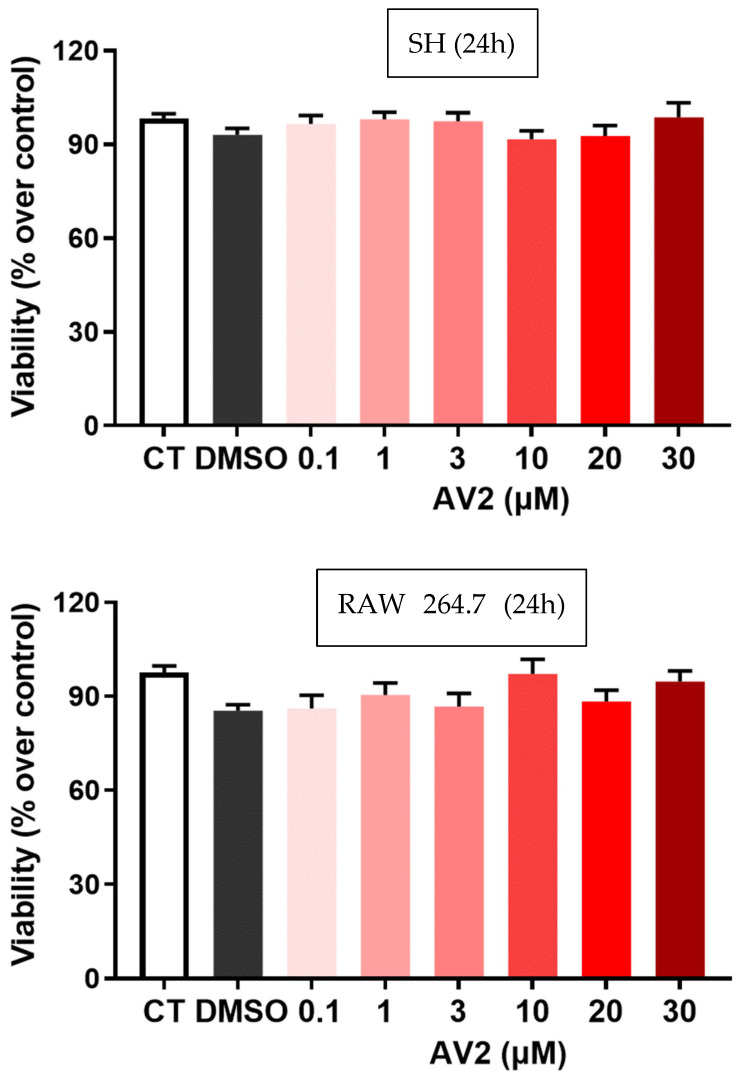
Cytotoxicity of AV2 (0.1 to 30 µM concentrations, in red scaling) after 24 h incubation in SH and RAW 264.7 cells. Cell viability was measured with fluorescein diacetate, and data were expressed as % over control, mean ± SEM (n = 5 repeated experiments). CT: control. No significant difference was observed (one-way ANOVA with Bonferroni post hoc test).

To investigate the biological profile of AV2, cell viability was evaluated in both SH and RAW 264.7 cell lines after treatment with the compound at concentrations ranging from 0.1 to 30 µM. The results showed the safe profile of AV2 as the compound did not modify SH or RAW 264.7 cell viability after 24 h of treatment (Figure 3).

#### 2.1.2. In Vitro Potential Cytoprotective Activity of AV2

**Figure 4:**
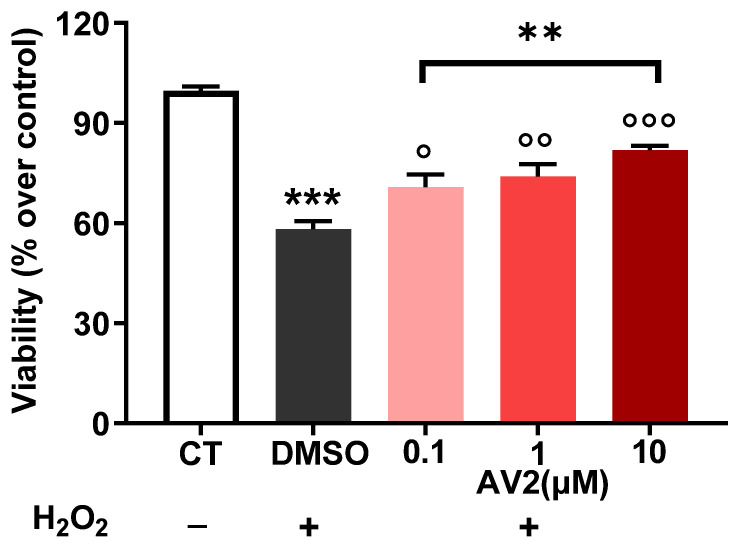
Cytoprotective activity of AV2 in vitro. AV2, at increasing concentrations (red scaling), reduced oxidative stress damage caused by H_2_O_2_ after 30 min of treatment in SH cells. Fluorescein diacetate assay was used to measure cell viability. CT: control, taken as 100%. ** *p* < 0.01, *** *p* < 0.001 vs. CT; ° *p* < 0.05, °° *p* <0.01, °°° *p* < 0.001 vs. DMSO+ H_2_O_2_; (one-way ANOVA *p* < 0.001; F = 71.96, with multiple comparisons).

In SH cells, AV2 at concentrations ranging from 0.1 to 10 µM reduced ROS formation triggered by H_2_O_2_ in a concentration-dependent manner, thus demonstrating its cytoprotective activity (Figure 4).

**Figure 5:**
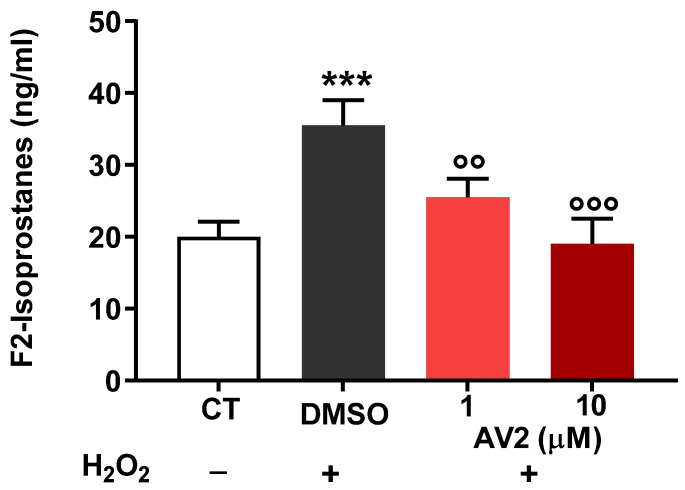
Concentration of total F2-isoprostanes measured in SH cells after oxidative stress generated by H_2_O_2_ in the absence (DMSO) or presence of AV2 (1–10 µM). CT: control. *** *p* < 0.001 vs. CT; °° *p* < 0.01; °°° *p* < 0.001 vs. DMSO; (one-way ANOVA, *p* < 0.05, F = 12.7, with multiple comparisons).

The protective effects of AV2 against oxidative stress damage in SH cells prompted us to look at F2-isoprostane levels. In oxidative conditions, the levels of F2-isoprostanes significantly increased (Figure 5); however, AV2 significantly decreased F2-isoprostanes in a concentration-dependent manner.

**Figure 6:**
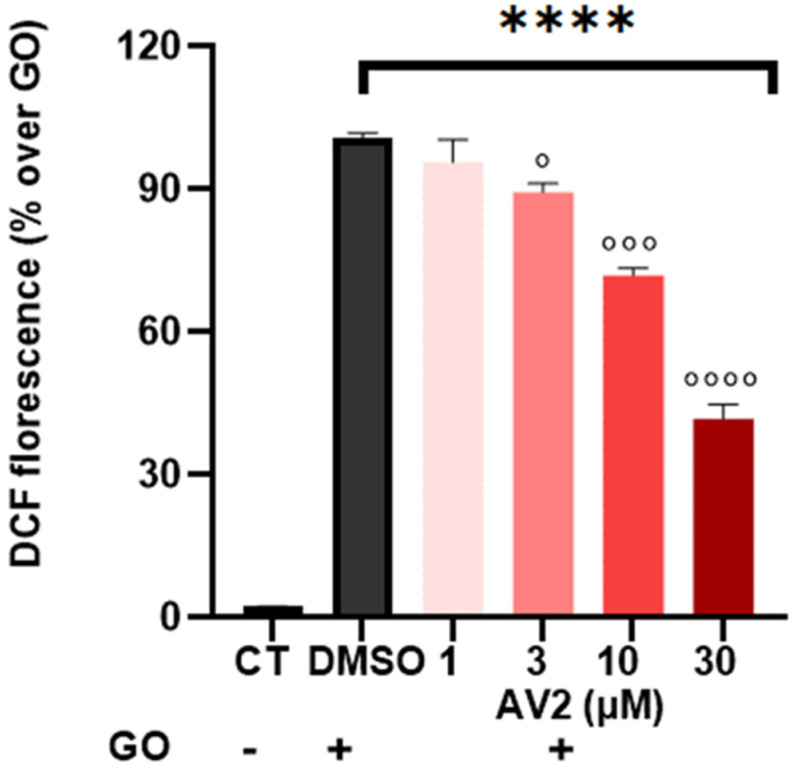
Cytoprotective activity of AV2 in vitro. Intracellular ROS formation in RAW264.7 cells produced by GO (DMSO), taken as 100%. CT: control. **** *p* < 0.0001 vs. CT; ° *p* < 0.05, °°° *p* < 0.001; °°°° *p* < 0.0001 vs. DMSO + GO; (one-way ANOVA *p*< 0.05; F = 4.12, with multiple comparisons).

The effect of AV2 on intracellular oxidant levels in RAW264.7 cells, evaluated using DCFH-DA as a fluorescent probe after glucose oxidase (GO) stimulation, is shown in Figure 6. AV2 reduced GO-induced ROS generation in a concentration-dependent manner, with the highest activity at 30 µM (Figure 6).

#### 2.1.3. Anti-Inflammatory Effects of AV2

**Figure 7:**
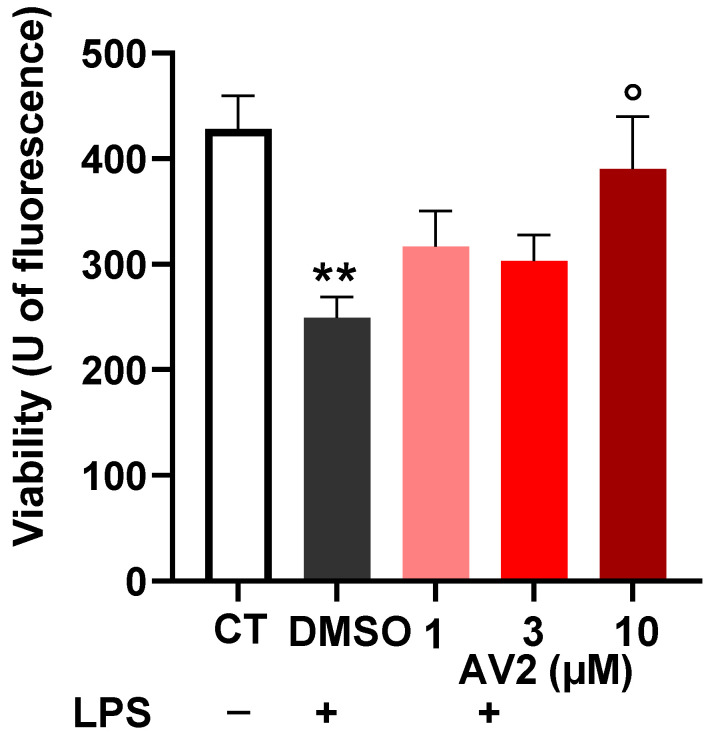
Effects of AV2 on LPS-induced damage in RAW264.7 cells. Cells were treated with LPS (1 μg/mL) in the absence (DMSO) or presence of AV2 at the indicated concentration for 24 h. Each bar represents the mean ± SEM calculated from three independent experiments. The significance was determined by one-way ANOVA (*p* < 0.0001, F = 7.36) with multiple comparisons. ** *p* < 0.01 vs. CT; ° *p* < 0.05 vs. DMSO + LPS. LPS: lipopolysaccharide. CT: control.

RAW264.7 cells were incubated with lipopolysaccharide (LPS) in the presence or absence of AV2, and the expression of TNF-α in the culture supernatant of cells was quantified. LPS (0.1 ug/mL) treatment significantly decreased the cell viability (DMSO); AV2 at 10 µM concentration significantly counteracted this effect, restoring control levels (Figure 7).

**Figure 8:**
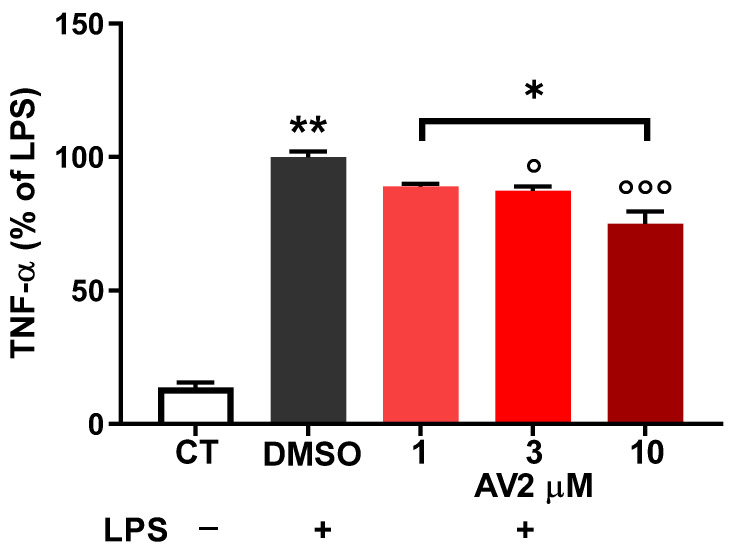
Effects of AV2 on LPS-induced TNF-α production in RAW264.7 cells. RAW264.7 cells were treated with LPS (1 µg/mL) in the absence (DMSO) (taken as 100%) or presence of AV2 for 24 h. TNF-α in the cultured supernatant was measured by ELISA. Each bar represents the mean ± standard deviation calculated from three independent experiments. The significance was determined by one-way ANOVA (F = 161.4; *p* < 0.0001), followed by multiple comparisons. * *p* < 0.05, ** *p* < 0.01 vs. CT; ° *p* < 0.05; °°° *p* < 0.001 vs. DMSO + LPS. CT, control; LPS, lipopolysaccharide; TNF-α, tumor necrosis factor-α.

Moreover, as shown by ELISA, the expression of TNF-α was relatively low in untreated control cells while it was markedly increased upon exposure to LPS alone. Pre-treatment with AV2 at 3 and 10 µM significantly reduced TNF-α production in LPS-stimulated RAW264.7 cells (Figure 8).

#### 2.1.4. Measurement of [Ca^2+^]i in Whole Cell Suspensions

**Figure 9:**
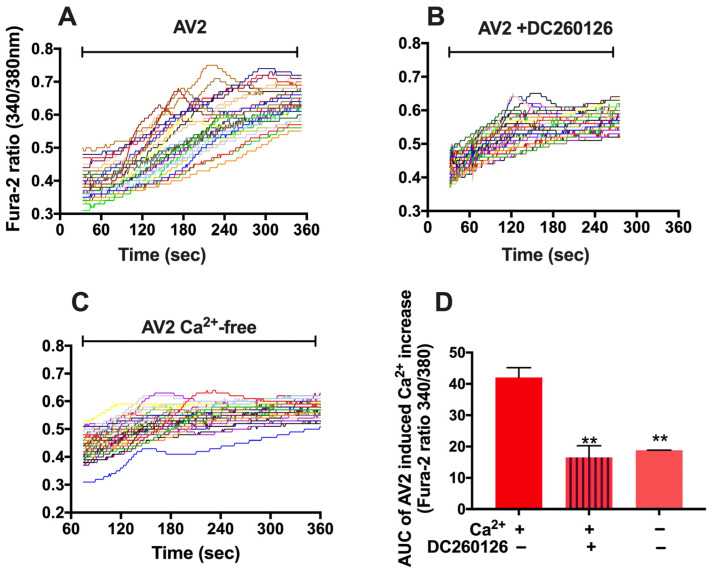
AV2 induces Ca^2+^ increase in SH cells. Intracellular Ca^2+^ was monitored over time in cells loaded with the Ca^2+^ indicator Fura-2 and stimulated withAV2 (10 µM) in a medium containing 1 mM CaCl_2_. The cells were stimulated with AV2 alone (**A**) or after 15 min pre-incubation with the FFAR1 antagonist DC260126 (**B**) or in Ca^2+^-free conditions (**C**). Each color represents a single cell response. In (**D**), the data of each bar represent the AUC of the mean ± SEM, calculated from three independent experiments. ** *p* < 0.01 vs. AV2 + Ca^2+^.

Measurement of intracellular calcium levels is a widely used method to detect FFARs activation by a ligand [21]. In our experiments, changes in intracellular calcium levels were measured in SH cells following cell stimulation by AV2 (10 µM) in a buffer containing 1 mM CaCl_2_ or in Ca^2+^-free conditions. AV2 induced a substantial transient increase in intracellular calcium that was largely reduced in the absence of calcium and in cells preincubated for 15 min with the FFAR1 antagonist DC260126. Data are expressed as change in ratio (340/380 nm) values and are from three different experiments, each with at least 20 cells (Figure 9).

### 2.2. In Vivo Experiments

#### 2.2.1. AV2 Anti-Inflammatory Effects

**Figure 10:**
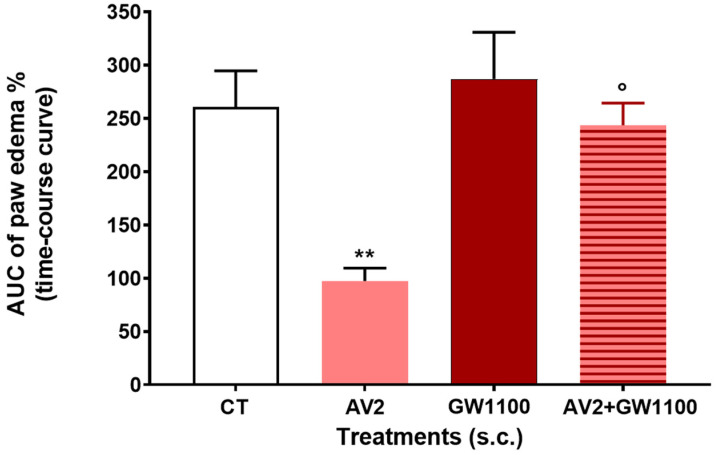
AV2, GW1100, and AV2 + GW1100 effects on paw edema induced by zymosan A. AV2 was administered at the dose of 100 μg/mouse, whereas GW1100 was administered at the dose of 10 μg/mouse 15 min before zymosan A; in the antagonism experiments, GW1100 was administered together with AV2, 15 min before zymosan A. Data were analyzed using one-way ANOVA, followed by Tukey’s multiple-comparisons test. F_3,24_ = 11.06, *p* < 0.0001; ** *p* < 0.01 vs. control (CT)-treated animals; ° *p* < 0.05 vs. AV2. (CT = DMSO/saline 1:5 *v*/*v*), n = 7.

The administration of a solution of zymosan A, a polysaccharide mixture from *Saccharomyces cerevisiae*, was used to induce an inflammatory response. The results of these experiments are shown in Figure 10.

To evaluate whether AV2 inhibited zymosan A-induced paw edema, mice were pre-treated with AV2 (100 μg) subcutaneously (s.c.) in the hind paw 15 min before zymosan A s.c. injection, and edema was assessed from 1 to 24 h after the stimulus. The results indicated that the formation of edema was significantly inhibited by AV2. The s.c. administration of the FFAR1 antagonist GW1100 did not alter per se the inflammatory properties of zymosan A, while GW1100 was able to reverse the anti-inflammatory effect of AV2 (Figure 10).

#### 2.2.2. AV2 Antinociceptive Effects

**Figure 11:**
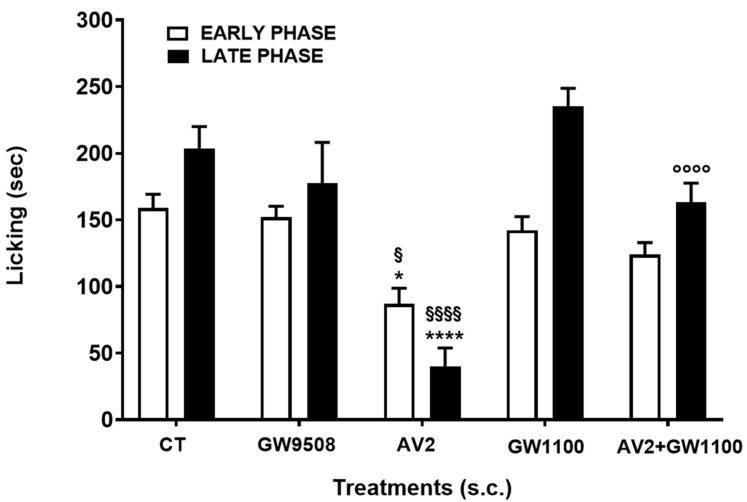
GW9580, GW1100, AV2, and AV2 + GW1100 effects in the formalin test after s.c. administration. GW9580 and AV2 were administered at the dose of 100 μg/mouse, whereas GW1100 was administered at the dose of 10 μg/mouse 15 min before formalin; in the antagonism experiments, GW1100 was simultaneously administered with AV2, 15 min before formalin. Data were analyzed using one-way ANOVA, followed by Tukey’s multiple-comparisons test. Early phase: F_4,30_ = 8.316, *p* < 0.0001. Late phase: F_4,30_ = 15.41, *p* < 0.0001; * *p* < 0.05 and **** *p* < 0.0001 vs. control (CT)-treated animals; § *p* < 0.05 and §§§§ *p* < 0.0001 vs. GW9508; °°°° *p* < 0.0001 vs. AV2. (CT= DMSO/saline 1:5 *v*/*v*), n = 7.

The formalin test was used to investigate the possible antinociceptive effects of AV2. In all experiments, the antinociceptive effect of AV2 was compared to that induced by GW9508, an FFAR1 partial agonist. Furthermore, to verify whether the effects of AV2 were dependent on an interaction between the compound and FFAR1, AV2 was co-administered together with the FFAR1 antagonist GW1100. As reported in Figure 11, AV2 s.c. administration in the hind paw at the dose of 100 μg/mouse was able to reduce licking activity in both the early and late phase of the formalin test, whereas GW9508 did not change licking duration. When AV2 was co-administered together with GW1100, the AV2-mediated antinociceptive effect was significantly abolished in the late phase and slightly reduced in the early phase of the formalin test.

**Figure 12:**
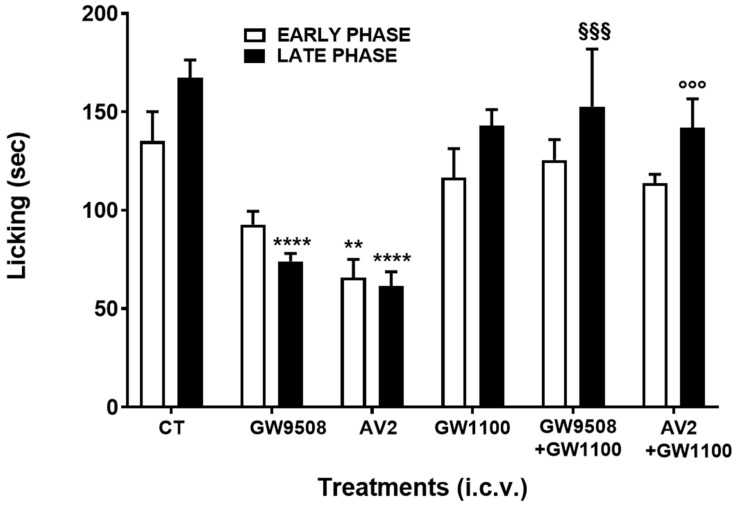
GW9508, GW1100, AV2, GW9508 + GW1100, and AV2 + GW1100 effects on the formalin test after i.c.v. administration. All compounds were administered i.c.v. at the dose of 1 μg/mouse 15 min before formalin. In the antagonism experiments, GW1100 was simultaneously administered with GW9508 or AV2. Data were analyzed using one-way ANOVA, followed by Tukey’s multiple-comparisons test. Early phase: F_5,36_= 5.428, *p* < 0.001. Late phase: F_5,36_= 9.101, *p* < 0.0001; ** *p* < 0.01 and **** *p* < 0.0001 vs. control (CT)-treated animals; §§§ *p* < 0.001 vs. GW9508; °°° *p* < 0.001 vs. AV2. (CT= DMSO/saline 1:5 *v*/*v*), n = 7.

The effect of AV2 in the formalin test after intracerebroventricular (i.c.v.) administration was then investigated, and the results are reported in Figure 12. GW9508 administered i.c.v. at the dose of 1.0 μg/mouse significantly decreased licking duration in the late phase but not in the early phase of the formalin test (Figure 12). AV2 administered i.c.v. at the dose of 1.0 μg/mouse was able to reduce licking duration in both the early and late phase of the test. The FFAR1 antagonist GW1100 (1 µg/mouse), when simultaneously administered i.c.v. either with GW9508 or with AV2, reduced the antinociceptive effects of both compounds, as shown in Figure 12.

## 3. Discussion

The main result of the present study is the clear evidence of the anti-inflammatory and antinociceptive effect of the quercetin-3-oleate (AV2) compound. Indeed, in all tested models, obtained either with in vitro or in vivo studies, AV2 showed a clear modulatory role of FFAR1, well known to modulate inflammation and pain.

As a first outcome, the in vitro safety profile of quercetin-3-oleate AV2 was shown in both neuroblastoma (SH) and macrophage cell lines (Raw 264.7). SH cells were chosen since neurons are very sensitive to inflammation and oxidative stress [22,23] and can have an important role in pain, whereas Raw 264.7 cells were selected as macrophages and are crucial for the host’s defense against inflammatory processes. In these cell lines, increased levels of cellular ROS were generated using hydrogen peroxide or through glucose-oxidase (GO) stimulation, respectively. When cells are exposed to a prolonged imbalance in ROS concentrations, impairment of cellular functions occurs, triggering several chronic and degenerative diseases, as well as an increased risk of cancer development [24,25]. AV2 was found to play an important protective role against a possible increase in ROS; interestingly this effect was concentration-dependent. Our results agree with the previous literature reports that ROS production was reduced in the presence of FFAR1 agonists [26,27,28]. In addition to ROS, other endogenous compounds, such as eicosanoids, and, in particular, F₂-isoprostanes, are mediators of inflammation and key factors of numerous acute and chronic inflammatory-based diseases [29]. In SH cells, the anti-inflammatory role of AV2 was confirmed by the significant reduction of F2-isoprostanes levels. Moreover, our results showed that AV2 inhibited the release of TNF-α induced by LPS in RAW 264.7 cells. This supports other studies [30,31,32] showing that activation of FFAR1 is able to induce anti-inflammatory effects by reducing the expression and levels of pro-inflammatory cytokines. In particular, in a recent study, 17,18-epoxyeicosatetraenoic acid, an FFAR1 agonist, significantly inhibited TNF-α-induced production of IL-6, IL-8, and mucin from cultured human airway epithelial cells, and these anti-inflammatory effects were abolished by the selective FFAR1 antagonist GW1100 [33]. On the whole, these data suggest that the role of FFAR1 activation in inflammatory processes may also depend on modulation of the levels of or effects induced by pro-inflammatory cytokines.

Finally, in agreement with another study [34] showing that FFAR1 activation enhances Ca^2+^ release from the endoplasmic reticulum by activating inositol 1,4,5-triphosphate receptors, in SH cells, AV2 induced a strong transient increase in intracellular calcium, an effect counteracted by the presence of the FFAR1 antagonist DC260126.

Our in vitro results highlighted the anti-inflammatory properties of quercetine-3-oleate (AV2) through the activation of FFAR1. This led us to study the in vivo AV2 anti-inflammatory and antinociceptive effects by means of the zymosan A-induced paw edema test and the formalin test, respectively. Importantly, as expected, in mice pre-treated with AV2, zymosan-induced edema was significantly inhibited, and the involvement of FFAR1 was clearly confirmed by the FFAR1 antagonist GW1100.

Zymosan A, a yeast cell wall extract, is a phagocytic stimulant which induces inflammatory responses characterized by an increase in vascular permeability and cellular infiltration, leading to edema [35,36]. The anti-inflammatory effects of some FFAR1 agonists (i.e., GW9508 and AS2034178) have been shown in several animal disease models and in inflammatory-based pathologies for which effective therapies have not yet been found [37,38,39]. With the present experiments, we have confirmed the FFAR1-induced effect by administering a product obtained from natural compounds, AV2.

Regarding the antinociceptive activity, this study demonstrates, for the first time, that AV2 strongly inhibits formalin-induced licking in both phases of the test and regardless of the route of administration (subcutaneously or intracerebroventricularly). Interestingly, the FFAR1 antagonist GW1100 reversed the AV2-induced antinociceptive effects in the late phase of the formalin test. The antinociceptive efficacy of AV2 was obtained via i.c.v. injection but also by subcutaneous administration, suggesting that FFAR1 receptors are situated in the periphery in cells involved in local inflammation. Chronic inflammation and inflammatory-based pain depend on changes in the tissue microenvironment in which increased levels of inflammatory mediators from immune cells, glial cells, and neurons modulate nociceptors in the central nervous system [40]. In this context, the connection between ROS and inflammation pathways is clear. When cells are exposed to a prolonged imbalance in ROS concentrations, impairment of cellular functions occurs, triggering several chronic and degenerative diseases, as well as an increased risk of cancer development [40].

The expression of FFAR1 protein was observed in brain areas such as the olfactory bulb, striatum, hippocampus, midbrain, hypothalamus, medulla oblongata, cerebellum, and cerebral cortex, as well as in the spinal cord, and some of these areas are involved in nociceptive processes [13]. After intracerebroventricular administration, the endogenous FFAR1 agonist docosahexaenoic acid (DHA) and GW9508 reduced formalin-induced pain behavior and attenuated complete Freund’s adjuvant-induced mechanical allodynia and thermal hyperalgesia [14]. Further evidence suggests that the antinociceptive effects of FFAR1 agonists may be dependent on the regulation of the descending pain control system via FFAR1-labeled neurons in the rostral ventromedial medulla or the locus coeruleus [12]. However, other mechanisms can explain the effects of FFAR1 and its agonists on nociception. Intrathecal injection of GW9508 dose-dependently reduced ipsilateral mechanical allodynia and thermal hyperalgesia in mouse models of inflammatory and neuropathic pain, and the FFAR1 antagonist GW1100 almost completely reversed these effects [37]. Patch-clamp recordings from spinal cord slices showed that the bath application of GW9508 significantly reduced the frequency of spontaneous excitatory postsynaptic currents in substantia gelatinosa neurons in pain models [37]. Interestingly, FFAR1 is not only involved in pain control but also in the transition from acute to chronic pain [38] and in chronic social defeat stress-induced pain prolongation [39,41].

In conclusion, quercetin-3-oleate AV2 is an ester easily obtained from two dietary compounds and endowed with antioxidant, anti-inflammatory, and antinociceptive activities. The efficacy of AV2 in alleviating inflammation and pain through FFAR1 has been demonstrated in vivo for the first time. These findings indicate that this derivative of the flavonoid quercetin could be a promising therapeutic molecule for the clinical management of inflammatory diseases and related pain conditions. In view of the potential therapeutic use of AV2, further studies to verify its effectiveness when administered orally would be of interest.

## 4. Materials and Methods

### 4.1. In Vitro Studies

#### 4.1.1. Cell Cultures and AV2 Treatment

Human immortal neuroblastoma SH cells and murine macrophagic RAW 264.7 cells (ATCC, Rockville, MD, USA) were grown under standard conditions. AV2 was dissolved in dimethyl sulfoxide (DMSO) and then diluted with cell culture medium to the desired concentrations. The final DMSO concentration did not exceed 0.1% (*v*/*v*), a concentration at which it did not impact cell viability. Cells were treated with AV2 at increasing concentrations for 24 h in all the assays described below.

#### 4.1.2. Cell Viability Assay

SH or RAW 264.7 cells in 96-well plates were incubated with AV2 (0.1, 1, 3, 10, and 30 μM) for 24 h, after which cell viability was assessed with fluorescein diacetate added to the cells at a final concentration of 10 µg/mL. Fluorescence was examined with a Fluoroskan Ascent fluorimeter (ThermoLabsystems) (355 nm excitation and 460 nm emission) for fluorescein diacetate. Fluorescence of vehicle- or medium-treated cells (control, CT) was taken as 100% of viability [42,43].

#### 4.1.3. Oxidative Stress and ROS Formation

To evaluate AV2-induced protection against oxidative stress, the following procedures were carried out.

(1) Following the published procedure [42,43], SH cells and RAW 264.7 cells were treated with medium or AV2 (1–30 μM) for 24 h. Then, SH cells were incubated in a 96-well plate for 30 min with H_2_O_2_ (0.5 mM final concentration); RAW 264.7 cells were loaded with DCFH-DA (20 µM for 30 min at 37 °C) and afterward with GO (1 U/mL, 15 min). In the presence of oxygen, GO catalyzes the oxidation of β-D-glucose to D-gluconic acid, producing H_2_O_2_ as a by-product. H_2_O_2_ causes the formation of ROS, determined by the cell permeant dye 2′,7′-dichlorofluorescein diacetate (DCFH-DA) [43]. The intracellular formation of 2′,7′-dichlorofluorescein by ROS was detected after 15 min at 485 nm (excitation) and 530 nm (emission) with a Fluoroskan Ascent fluorimeter (ThermoLabsystems). DCF produced by GO was taken as 100%.
(2) F2-isoprostanes (F2-IsoPs) were evaluated to assess lipid peroxidation being produced by oxidative damage of membrane arachidonic acid. F2-IsoPs were measured by gas chromatography/negative-ion chemical ionization/tandem mass spectrometry (GC/NICI-MS/MS), as previously described [42]. Briefly, SH cells were lysed, and 100 µM butylated hydroxytoluene (BHT) was added. Afterward, each sample was incubated with 1 N KOH at 45 °C for 45 min. At the end of the incubation, samples were acidified using 1 N HCl, and tetra-deuterated prostaglandin F2α (PGF2α-d4) (500 pg) was added before extraction in the presence of ethyl acetate (10 mL). The lipid extract obtained was purified by solid-phase extraction using an NH_2_ cartridge. The eluates obtained from the purification were subjected to two derivatization steps prior to GC/NICI-MS/MS analysis in which the ion at m/z 299, derived from 15-F2t-IsoP (one of the most common isomers of F2-IsoP), was detected [42]. The authentic molecule 15-F2t-IsoP (Cayman Chemical, item no. 16350) was used for calibration.

#### 4.1.4. Inflammation and Cytokine Measurement

Confluent RAW264.7 cells in 96-well plates were incubated with AV2 (1–30 µM) for 24 h and then co-incubated with 0.10 µg/mL LPS, and fluorescence was measured. The cytokine TNF-α released from LPS-treated cells was measured in cell culture supernatants using a TNF-α ELISA KIT (Thermo Fisher, Waltham, MA, USA), following the manufacturer’s instructions.

#### 4.1.5. Single-Cell Calcium Imaging Experiments on Adherent Cells

To evaluate changes in cytosolic-free calcium concentration, cells were plated on eight-well glass chamber slides (Sarstedt, Nümbrecht, Germany) at the density of 4 × 10^4^ cells/chamber; adherent cells were then loaded with the fluorescent calcium indicator Fura-2 acetoxymethyl ester (Fura-2-AM), 3 µM, in a medium containing 140 mM NaCl, 5.4 mM KCl, 1 mM MgCl_2_, 1 mM CaCl_2_, 10 mM glucose, 1% bovine serum albumin (BSA), and 15 mM Hepes buffer, pH 7.4, to evaluate changes in cytosolic-free calcium concentration. After Fura-2 loading for 30 min at room temperature, cells were maintained in the same medium without Fura-2-AM and BSA. For Ca^2+^ measurements, a fluorescence microscope with an imaging system was used. The digital fluorescence-imaging microscopy system was mounted on a Nikon Diaphot 300 inverted microscope (Nikon, Tokyo, Japan). Fluorescence images were collected through a Nikon oil immersion 40X/13 numerical aperture objective and acquired by a cooled charge-coupled device camera (Photometrics, Roper Scientific, Trenton, NJ, USA) and a MetaFluor imaging system (Universal Imaging, Downingtown, PA, USA). The ratio of the fluorescence at both 340 and 380 excitation wavelengths was monitored and acquired [20]. To quantify the Ca^2+^ response to AV2, the area under the curve (AUC) of the Fura-2 ratio in 4 min after the addition of AV2 was measured [44].

### 4.2. In Vivo Experiments

Male CD-1 mice (Harlan, Correzzana, Italy) aged 3–4 weeks (25 g) were used for all experiments. Mice were housed in colony cages, under standard conditions of light, temperature, and relative humidity, for at least 1 week before the start of experimental sessions. All experiments were performed according to Legislative Decree 27/92 and approved by the local ethics committee (Approval number 198/2013-B).

#### 4.2.1. Surgery

To carry out intracerebroventricular (i.c.v.) administration, the following procedure was performed as described previously [45]. Briefly, the mice were anesthetized with intraperitoneal (i.p.) ketamine (80 mg/kg) and xylazine (10 mg/kg) and implanted unilaterally with a steel guide cannula projecting to the lateral ventricle using stereotactic coordinates (bregma, anteroposterior, −0.3 mm; lateral, 2 mm; depth, 2 mm). A dummy cannula was then inserted into the guide cannula and fixed with cap nuts. Skull screws and dental cement were used to anchor the cannula to the skull. The mice were allowed to recover after surgery for at least 7 days. The injection was performed with a 25 mL Hamilton microliter syringe fitted with a 26-gauge needle, which was connected via a polyethylene tube to an internal cannula. At the end of the experiments, the location of the cannula was randomly verified histologically by sectioning the brain and staining with cresyl violet.

#### 4.2.2. Substances

Quercetin-3-oleate (AV2) was obtained as previously reported [18]. GW1100 (FFAR1 antagonist) was purchased from Cayman Europe OÜ (Tallinn, Estonia). GW9508 (FFAR1 partial agonist), dimethyl sulfoxide (DMSO), and formalin were purchased from Merk Life Science S.r.l. (Milan, Italy). Unless otherwise stated, all the other reagents were purchased from Carlo Erba (Milan, Italy).

On the day of the experiment, AV2, GW9508, and GW1100 were dissolved in 100% DMSO. Solutions were freshly prepared using saline containing 0.9% NaCl and DMSO in the ratio DMSO/saline 1:5 *v*/*v* every experimental day. These solutions were injected subcutaneously (s.c.) into the dorsal surface of the mouse hind paw or intracerebroventricularly (i.c.v.) at a volume of 20 or 10 µL/mouse, respectively. The drugs under investigation were injected s.c. at doses of 10 or 100 mg/mouse or i.c.v at the dose of 1.0 μg/mouse, based on previous publications [14].

#### 4.2.3. Zymosan A-Induced Paw Edema

One week after arrival, mice belonging to a cage were transferred to a dedicated experimental room to be tested. Each mouse received a subcutaneous injection (20 μL/paw) of zymosan A (2.5% *w*/*v* in saline) into the dorsal surface of the right hind paw, as previously described [45,46,47,48]. Paw volume was measured three times prior to the injection and then at 1, 2, 3, 4, 5, and 24 h post-injection, using a hydroplethysmometer (Ugo Basile, Italy). The increase in paw volume was calculated as a percentage. Changes were plotted and analyzed using GraphPad Prism software, version 9.2.0 (GraphPad Software Inc., San Diego, CA, USA). Drugs were administered subcutaneously into the dorsal surface of the right hind 15 min before zymosan injection.

#### 4.2.4. Formalin Test

One week after arrival, mice were singly transported in the experimental room and, as previously described [49], were placed in a Plexiglas observation cage (30 × 14 × 12 cm) one hour before formalin injection to acclimate. Then, a dilute solution of formalin (1%, 20 μL/paw) was injected subcutaneously into the dorsal hind paw. Following the formalin injection, mice were returned to the Plexiglas observation cage, and behavior was continuously recorded using a stopwatch at 5-minute intervals over a 40-minute testing period. The total time (in seconds) spent licking or biting the paw during the early (0–10 min) and late (10–40 min) phases of the formalin test was recorded. Drugs were administered either subcutaneously or intracerebroventricularly 15 min before formalin injection.

### 4.3. Statistical Analysis

Data are expressed as mean ± SEM. Significant differences among the groups were evaluated with one-way ANOVA, followed by a multiple-comparisons test, as indicated. GraphPad prism software (version 9.2.0, GraphPad Software Inc., San Diego, CA, USA) was used for all the analyses. Statistical significance was set at *p* < 0.05. Data from cell viability, cytoprotective activities, the F2-isoprostane, and TNF-α levels were analyzed using one-way ANOVA, followed by a Bonferroni post hoc test. To analyze intracellular calcium levels, data were expressed as a change in ratio (340/380 nm) values, and the area under the curve (AUC) was calculated and analyzed using one-way ANOVA, followed by the Bonferroni post hoc test. Edema data were calculated as the percentage difference between the paw volume at each time point (1, 2, 3, 4, 5, and 24 h post-injection) and the baseline paw volume. Then, the area under the percentage curve was computed and analyzed using one-way ANOVA, followed by Tukey’s multiple-comparisons test. To determine the antinociceptive effect and statistical significance, formalin early- and late-phase data were tested with one-way ANOVA, followed by Tukey’s multiple-comparisons test. The data and statistical analysis comply with the recommendations on experimental design and analysis in pharmacology.

## Data Availability

Data is contained within the article or Supplementary Material.

## Supplementary Materials

The following are available online at https://www.mdpi.com/article/10.3390/ijms252111635/s1.
