# Supplementary material for: Anti-Inflammatory and Antinociceptive Properties of the Quercetin-3-Oleate AV2, a Novel FFAR1 Partial Agonist
Source: Int J Mol Sci. 2024 Oct 30;25(21):11635. doi: 10.3390/ijms252111635 (PMC11546106; doi:10.3390/ijms252111635)
Supplement: Supplementary file 1 [file ijms-25-11635-s001.zip › ijms-3256966-supplementary.pdf]

“Supplementary Materials”

Anti-inflammatory and antinociceptive properties of the quercetin-3-oleate AV2, a novel FFAR1 partial agonist.

Federica Pessina 1,*†, Ilenia Casini2†, Alessandra Gamberucci1, Gabriele Carullo3, Cinzia Signorini1, Antonella Brizzi3,* Francesca Aiello4, Anna Maria Aloisi 2 ,‡ and Stefano Pieretti 5,‡

Table of contents

S1-S6 Prediction of Pharmacokinetic Properties of quercetin-3-oleoyl derivative AV2.

TABLE S1 Table S1. Expected Chemical properties

TABLE S2 Table S2. Expected ADME properties.

.

Table S1. Expected Chemical properties

Descriptor	Value
Molecular Weight	566.691
LogP	8.2253
#Rotatable Bonds	17
#Acceptors	8
#Donors	4
Surface Area	240.468

Table S2. Expected ADME properties

Property	Model Name	Predicted Value	Unit
Absorption	Water solubility	**-3.557**	Numeric (log mol/L)
Absorption	Caco2 permeability	**-0.122**	Numeric (log Papp in 10^{-6} cm/s)
Absorption	Intestinal absorption (human)	**88.258**	Numeric (% Absorbed)
Absorption	Skin Permeability	**-2.735**	Numeric (log Kp)
Absorption	P-glycoprotein substrate	**Yes**	Categorical (Yes/No)
Absorption	P-glycoprotein I inhibitor	**Yes**	Categorical (Yes/No)
Absorption	P-glycoprotein II inhibitor	**Yes**	Categorical (Yes/No)
Distribution	VDss (human)	**-0.696**	Numeric (log L/kg)
Distribution	Fraction unbound (human)	**0.167**	Numeric (Fu)

Property	Model Name	Predicted Value	Unit
Distribution	BBB permeability	**-2.067**	Numeric (log BB)
Distribution	CNS permeability	**-2.945**	Numeric (log PS)
Metabolism	CYP2D6 substrate	**No**	Categorical (Yes/No)
Metabolism	CYP3A4 substrate	**Yes**	Categorical (Yes/No)
Metabolism	CYP1A2 inhibitior	**No**	Categorical (Yes/No)
Metabolism	CYP2C19 inhibitior	**Yes**	Categorical (Yes/No)
Metabolism	CYP2C9 inhibitior	**Yes**	Categorical (Yes/No)
Metabolism	CYP2D6 inhibitior	**No**	Categorical (Yes/No)
Metabolism	CYP3A4 inhibitior	**No**	Categorical (Yes/No)
Excretion	Total Clearance	**0.291**	Numeric (log ml/min/kg)

Property	Model Name	Predicted Value	Unit
Excretion	Renal OCT2 substrate	**No**	Categorical (Yes/No)
Toxicity	AMES toxicity	**No**	Categorical (Yes/No)
Toxicity	Max. tolerated dose (human)	**0.328**	Numeric (log mg/kg/day)
Toxicity	hERG I inhibitor	**No**	Categorical (Yes/No)
Toxicity	hERG II inhibitor	**Yes**	Categorical (Yes/No)
Toxicity	Oral Rat Acute Toxicity (LD50)	**2.21**	Numeric (mol/kg)
Toxicity	Oral Rat Chronic Toxicity (LOAEL)	**2.576**	Numeric (log mg/kg_bw/day)
Toxicity	Hepatotoxicity	**Yes**	Categorical (Yes/No)
Toxicity	Skin Sensitisation	**No**	Categorical (Yes/No)
Toxicity	*T.Pyriformis* toxicity	**0.285**	Numeric (log ug/L)

Property	Model Name	Predicted Value	Unit
Toxicity	Minnow toxicity	**-2.361**	Numeric (log mM)
